# Bioinformatics—an entry-level avenue for biomedical research in Nepal

**DOI:** 10.3389/fgene.2014.00042

**Published:** 2014-03-19

**Authors:** Yadav Sapkota, Sudip Subedi

**Affiliations:** ^1^Department of Genetics and Computational Biology, QIMR Berghofer Medical Research InstituteBrisbane, QLD, Australia; ^2^Department of Oncology, University of AlbertaEdmonton, AB, Canada

**Keywords:** bioinformatics, computer science, Nepal, biotechnology, biomedical sciences

Nepal is a landlocked country blessed with stunning natural bounty. The nation still caught amidst process of transformation and restructuring is recuperating after an excruciating decade of civil war brunt of which mostly bore by its premature infrastructure. Nepal has several vital sectors categorized into top-priority for the revival process. It is saddening that “Science and Technology” does not make amongst the top few (Guo, 2008). Particularly, biomedical research has always been failing to catch up, in part due to astronomical costs of resources this form of research demands for. This, by no means, implies that the country lacks motivated scientists and trainees who carry in them the potential to contribute to the science in global arena.

Meanwhile, a rather “unconventional” area of biomedical research, bioinformatics, can be an alternative area of focus for the scientific community in Nepal—a discipline that aims to address the biological problems using computer software and simulations (Searls, 2010; Hogeweg, 2011). Individuals trained in bioinformatics are overwhelmingly in high demand worldwide and the surge for these highly skilled professionals is exponentially increasing in the post-genomic era (Wu, 2001; Yu et al., 2004), especially after the completion of the Human Genome Project and subsequent advances in high-throughput technologies to study the human genome.

Given the fact that the discipline of computer science has excelled tremendously in the past decade in Nepal and its financial feasibility relative to the wet-laboratories, it definitely looks like a viable option for the research enthusiasts. There are a few academic institutions in Nepal that are involved in some aspects of bioinformatics researches but these are largely limited to formal educational training; real-life application is by far lacking (Nepal Academy of Science and Technology and Ministry of Environment, 2008). The Department of Biotechnology at Kathmandu University, established in 2003, has been offering introductory courses on bioinformatics, genomics, and proteomics as a part of its undergraduate degree in biotechnology. A proper bioinformatics laboratory consisting of more than 25 decently configured computers with high-speed internet has been established, where students are provided with basic hands-on trainings on open-source bioinformatics tools and software. Similarly, the pioneer university of Nepal, Tribhuvan University, has also started teaching fundamental concepts in bioinformatics-related areas for students enrolled in its postgraduate degree in biotechnology. Furthermore, academic institutions such as Himalayan White House International College, Lord Buddha Education Foundation, SANN (Study of Ancient and New Nepal) International College and Universal Science College have incorporated bioinformatics in the curriculum of their undergraduate degree in biotechnology. However, at present, no university or academic institutions in Nepal run bioinformatics as an independent discipline either at undergraduate or postgraduate level (Nepal Academy of Science and Technology and Ministry of Environment, 2008). Besides, hundreds of biotechnology graduates with preliminary knowledge and trainings on bioinformatics are bound to go abroad for further education/trainings as in-house opportunities are limited.

More recently, an independent non-governmental organization, Center for Molecular Dynamics, has taken an initiative toward practical application of biotechnology (and/or bioinformatics) in Nepal, especially in molecular diagnostics and wildlife genomics. Nepal Tiger Genome Project is a unique example of such kind. While a more focused formal education and in-depth trainings on bioinformatics, perhaps through a few independent programs, are needed for producing technicians and experts in the field, real-life application of bioinformatics is equally essential for sustainable biomedical research in Nepal. Such an effort is also likely to attract international academic and research collaborations, especially while fast-growing economic giants like China and India are performing exceptionally well in the biomedical field. In this context, the Government of Nepal and the scientific fraternity within Nepal should instigate toward promotion of bioinformatics training and R&D and become a contributing member of the global scientific community.
